# Light therapy for multiple sclerosis-associated fatigue: Study protocol for a randomized controlled trial

**DOI:** 10.1097/MD.0000000000008037

**Published:** 2017-09-08

**Authors:** Farrah J. Mateen, Natalie C. Manalo, Sara J. Grundy, Melissa A. Houghton, Gladia C. Hotan, Hans Erickson, Aleksandar Videnovic

**Affiliations:** a Neurological Clinical Research Institute, Department of Neurology, Massachusetts General Hospital; b Harvard Medical School, Boston, MA; c Department of Brain and Cognitive Sciences, Massachusetts Institute of Technology, Cambridge, MA.

**Keywords:** CNS, demyelinating autoimmune disease, fatigue, light therapy, multiple sclerosis, nervous system diseases

## Abstract

**Background:**

Fatigue is the most commonly reported symptom among multiple sclerosis (MS) patients, more than a quarter of whom consider fatigue to be their most disabling symptom. However, there are few effective treatment options for fatigue. We aim to investigate whether supplemental exposure to bright white light will reduce MS-associated fatigue.

**Methods:**

Eligible participants will have clinically confirmed multiple sclerosis based on the revised McDonald criteria (2010) and a score ≥36 on the Fatigue Severity Scale (FSS). Participants will be randomized 1:1 to bright white light (10,000 lux; active condition) or dim red light (<300 lux; control condition) self-administered for 1 hour twice daily. The study will include a 2-week baseline period, a 4-week treatment period, and a 4-week washout period. Participants will record their sleep duration, exercise, caffeine, and medication intake daily. Participants will record their fatigue using the Visual Analogue Fatigue Scale (VAFS) 4 times every third day, providing snapshots of their fatigue level at different times of day. Participants will self-report their fatigue severity using FSS on 3 separate visits: at baseline (week 0), following completion of the treatment phase (week 6), and at study completion (week 10). The primary outcome will be the change in the average FSS score after light therapy. We will perform an intention-to-treat analysis, comparing the active and control groups to assess the postintervention difference in fatigue levels reported on FSS. Secondary outcome measures include change in global VAFS scores during the light therapy and self-reported quality of life in the Multiple Sclerosis Quality of Life-54.

**Discussion:**

We present a study design and rationale for randomizing a nonpharmacological intervention for MS-associated fatigue, using bright light therapy. The study limitations relate to the logistical issues of a self-administered intervention requiring frequent participant self-report in a relapsing condition. Ultimately, light therapy for the treatment of MS-associated fatigue may provide a low-cost, noninvasive, self-administered treatment for one of the most prevalent and burdensome symptoms experienced by people with MS.

## 1 Introduction

Fatigue is the most commonly reported symptom among people living with multiple sclerosis (pMS).^[1–4]^ More than a quarter of pMS state that fatigue is their most disabling symptom.^[5]^ Despite the impact of fatigue in MS, there are few effective treatment options. Many pMS seek nonpharmacological options for fatigue, but have limited evidence upon which to make important treatment decisions.

Light therapy (LT) has been associated with reduction of fatigue in other disorders, including Parkinson's disease,^[6]^ traumatic brain injury,^[7]^ seasonal affective disorder,^[8]^ and cancer-related fatigue.^[9]^ Fatigue may have multiple mechanisms in MS, including dysfunction of the dopaminergic neurons in the basal ganglia,^[10,11]^ endocrine dysfunction due to suppression of the hypothalamic pituitary adrenal axis,^[12]^ cytokine induction in a continuous, proinflammatory state of the brain,^[13]^ and/or reduced function of the frontal cortices.^[10]^ If mechanisms of fatigue are overlapping across disorders in which bright white LT has shown therapeutic benefits, LT may be an unrecognized therapeutic option for fatigue among pMS.

A prospective study conducted in Tasmania in 2013 of 198 pMS demonstrated that self-reported sunlight exposure was correlated with lower levels of fatigue.^[14]^ Although causality cannot be determined from that study, it suggests a possible therapeutic benefit of artificial light for fatigue in pMS. These beneficial effects of light on fatigue could be mediated through its direct alerting effects or through its effects as the main synchronizer of the circadian system.

Given the high burden of fatigue among pMS and the promising results of LT treatment for fatigue in other neurological disorders, we are performing a clinical trial to assess the efficacy of LT to treat MS-associated fatigue. We hypothesize that supplemental exposure to bright white light may be beneficial in MS-associated fatigue and improve the quality of life of pMS.

## 2 Methods

### 2.1 Trial design

**Figure 1:**
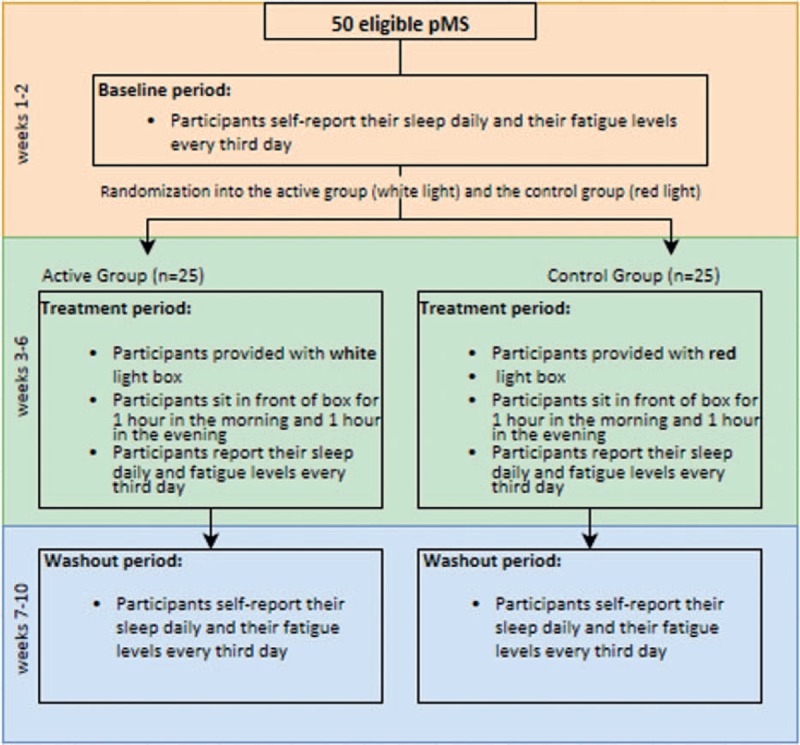
Light therapy for multiple sclerosis-associated fatigue study algorithm.

This is a 2-arm, randomized, controlled clinical trial of 50 adult pMS. Eligible participants will be assigned 1:1 to either control or active arms by the research coordinator using a computer-generated random number sequence (Fig. 1). The trial will investigate whether bright white LT 10,000 lux (active condition), compared with dim red LT <300 lux (control condition), will reduce self-reported fatigue among pMS when administered twice daily in 1-hour sessions for 4 consecutive weeks.

### 2.2 Study setting

The study will be centered at the Neurological Clinical Research Institute (NCRI) at Massachusetts General Hospital (MGH), Boston, MA. The intervention itself is at the home or office of the participant.

### 2.3 Recruitment

Participants are currently being recruited from a variety of sources. Many are recruited directly from the MS Clinic at MGH. We also sent letters to eligible pMS to assist with recruitment. We use MGH's Research Subject Volunteer Program (RSVP), a participant matching service, to identify participants with enthusiasm for the project. We have additionally posted advertisements through a clinical trials email that is sent out by the hospital and a posting on the National MS Society's public website (www.nationalmssociety.org). The trial is also listed on clinicaltrials.gov. If the pMS is a patient of the principal investigator, a research physician or study coordinator explains the study so that he or she does not feel obligated to participate.

### 2.4 Study participants

The participants will be ≥50 adult pMS who meet the inclusion and exclusion criteria.

### 2.5 Inclusion criteria

1. Above the age of 18 years and below the age of 70 years; male or female.
2. Diagnosed with relapsing remitting or progressive MS based on the revised McDonald Criteria (2010).^[15]^
3. The presence of fatigue defined as a score ≥36 on the Fatigue Severity Scale (FSS).^[16]^

### 2.6 Exclusion criteria

1. Change in antidepressant medication within the preceding 4 weeks.
2. Change in fatigue medication regimen within the preceding 4 weeks.
3. Change in MS disease-modifying therapy within the preceding 4 weeks.
4. Beck Depression Inventory II score >14.
5. Shift work.
6. Use of photosensitizing medication such as phenothiazines, chloroquine, amiodarone, or St. John's Wort.
7. The presence of eye trauma or acute optic neuritis within the preceding 3 months.
8. History of traumatic brain injury.
9. Probable (untreated) sleep apnea based on Berlin questionnaire.
10. Significant anemia, defined as hemoglobin <11 mg/dL.
11. History of mania.
12. Clinical MS relapse in the preceding 4 weeks.
13. Current pregnancy.
14. Known photosensitivity.
15. Other complicating illness preventing study completion.

### 2.7 Reasons to withdraw

Participants will be withdrawn from the study if they experience a clinical relapse requiring hospitalization or treatment with steroids during the study period, or if there is a change in disease modifying therapy for MS, depression, or fatigue during the study period. Participants will also be withdrawn from the study if they experience any life event that prohibits their participation. This is a safe treatment, and we anticipate minimal side effects.

### 2.8 Participant enrollment

A research coordinator will conduct a phone evaluation to screen for the exclusion and inclusion criteria, and if determined to be eligible, the participant will be scheduled for the baseline visit. After successful completion of the baseline visit, participants will be randomized using a computer-based random number generator. Participants will be assigned 1:1 to bright white LT (10,000 lux) or dim red LT (<300 lux) (Fig. 1). Dim red LT is a widely accepted control condition in clinical studies of LT. Randomization will occur at this time point to allow for light box distribution before initiation of the light therapy phase. This will reduce the burden on participants.

### 2.9 Baseline period

The baseline period is 2 weeks in duration and begins after the baseline visit. The baseline visit includes several surveys to ensure exclusion and inclusion criteria are upheld. Surveys are provided by research coordinators and filled out by participants. Surveys evaluating sleep quality, sleepiness, depression, and level of disability are used to assess these potential confounding variables: FSS,^[16]^ Beck Depression Inventory II (BDI),^[17]^ Pittsburg Sleep Quality Index (PSQI),^[18]^ Berlin questionnaire for obstructive sleep apnea,^[19]^ Epworth Sleepiness Scale (ESS),^[20]^ Multiple Sclerosis Quality of Life-54 (MSQOL-54),^[21]^ and the Kurtzke Extended Disability Status Scale (EDSS).^[22]^ The EDSS is administered by a neurologist who is a certified EDSS rater.

After the baseline visit, a chart review is conducted for each participant to collect clinical data on baseline neurological symptoms, imaging features, medical comorbidities, and serum 25-OH vitamin D levels. Studies in patients with MS-associated fatigue presently show inconclusive and conflicting results regarding the importance of the total serum vitamin D level with fatigue in MS.^[23–25]^ If the participant is seen elsewhere, we ask for a copy of their most recent medical note to confirm a diagnosis of MS.

During the baseline phase, participants record their daily sleep duration and exercise, as well as caffeine, alcohol, and medication intake. In addition, participants record their fatigue using the Visual Analogue Fatigue Scale (VAFS) 4 times every third day, starting after waking up, and repeating every 4 hours afterward until bedtime. The VAFS is a simple, validated 10-point scale ranging from 1 to 10 in which participants can report their fatigue as a snapshot at a particular moment.^[26]^ Participants will be provided with a logbook to record these measures. The research coordinator distributes the appropriate LT boxes (either bright white light or dim red light) to the participants after completion of the baseline visit to limit the number of required visits.

### 2.10 Treatment period

The treatment period is 4 weeks in duration and begins immediately after the 2-week baseline period. Participants not only continue to record their sleep and fatigue levels as in the baseline period, but also self-administer LT. Participants are instructed to sit in front of the box with their eyes approximately 18.5 in. from the light source. They use the box for 1 hour twice daily, in the morning starting 2 hours after awakening and in the evening starting 3 hours before bedtime. Eyes should be open while using LT, but participants do not have to look directly at the light and can eat, watch television, and so on during use. Participants may take home 1 or 2 boxes because some may use 1 box at work and 1 at home, for example. During the 4-week treatment period, participants record their use of LT as well as any side effects they are experiencing in the logbook provided.

At the end of the treatment period, participants have their first follow-up visit, during which they complete the FSS and the MSQOL-54 as well as discuss their subjective experiences with LT.

### 2.11 Washout period

The washout period is 4 weeks in duration and begins on week 7, immediately after the first follow-up visit. Participants continue to record their sleep and fatigue levels, and discontinue LT. At the end of the 4 weeks, participants have a second follow-up visit and complete a final FSS and MSQOL-54. Each participant is asked whether they believe the intervention had any effect on their fatigue and whether they believe the alternative therapy (bright white light or dim red light) would have been more or less beneficial. Participants in both arms will be asked the same questions.

### 2.12 Light therapy device

The light box used for LT is made by *The Sunbox Company*, located in Gaithersburg, MD. Each box is approximately 15.5” tall × 23” wide × 3.25” in height, and is designed to stand on a desk or tabletop. The light is delivered at a downward angle to maximize the effectiveness of the treatment. The box runs on 124 W and contains full spectrum 5000k 10,000 lux bulbs. The bright white LT is 10,000 lux and the dim red LT is <300 lux. Bright LT requires at least 2500 lux to be effective in the published literature.^[27]^

### 2.13 Blinding

Physicians who will be assessing outcomes and the statistician performing the study analyses will remain blinded to each participant's assigned condition (bright white light vs dim red light) throughout the study. Participants will not know if they were assigned to the active or control condition and will be reminded not to reveal their assignment to study investigators.

### 2.14 Study instruments

For survey descriptions, scoring, and eligibility in our clinical trial, see Table 1. Research coordinators will be available to discuss subjective and qualitative issues related to light therapy participation and completion.

**Table 1:**
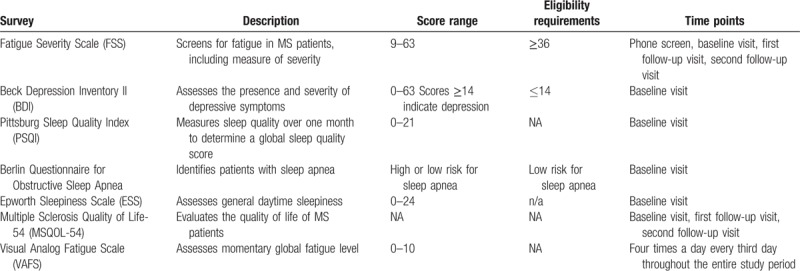
Survey instruments: descriptions and scoring in the LT clinical trial.

### 2.15 Estimated timeline of the study

Recruitment for this study began on April 1, 2017 and is anticipated to continue through December 2018. Anticipated recruitment is estimated to be approximately 4 new participants per month.

### 2.16 Participant remuneration

Subjects will be paid $125.00 for their participation in addition to receiving parking vouchers to cover parking expenses related to study visits.

### 2.17 Outcome measures

The primary outcome measure of this study is a change in the average FSS over the course of the 4-week treatment period. The FSS is administered at the baseline visit, the first follow-up visit, and the second follow-up visit. The secondary outcome measures are a change in the global VAFS scores after LT and a change in self-reported quality of life on the MSQOL-54. For participants who choose to withdraw from the study, we will inquire about their reasoning for doing so and report this qualitatively in the manuscript in regards to feasibility.

### 2.18 Data entry and storage

Data will be entered into password-protected Excel files by research coordinators and study staff. Only investigators will have access to the data. Any data point out of range will be verified with the original source. Set ranges are determined by staff who are not involved in data entry.

### 2.19 Statistical analysis

There are no available data on FSS in response to bright LT in MS to guide our sample size calculations. We will compare the mean change in FSS scores before initiation and after completion of LT, in an intention-to-treat analysis comparing the active and control condition groups. Participants who start the LT intervention in either arm will be included as intended-to-treat. To test the hypothesis of a proposed difference of ≥15 points on FSS, we will use the conventional values of *α* = 0.05 and *β* = 0.80 for 2-tailed tests of probability with equally sized groups. For a difference in ≥15 points, with an estimated SD of 15.2 points and mean of 45.9 points from preliminary data collection at the MGH MS Clinic, we require a minimum of 15 people in either group.

For global VAFS scores, frequencies will be used to describe baseline characteristics across treatment arms. *X*^2^ statistics or Fisher exact test will be used to compare the differences between groups. VAFS will be evaluated using a mixed-effects model, which accounts for correlation between repeated measurements. Participant logbooks will be used to generate summary statistics and to graphically display fatigue patterns throughout the day among pMS in both groups. Logbooks will also be used to qualitatively assess the feasibility, safety, and tolerability of LT.

## 3 Discussion

There is an unmet need to address fatigue in people with MS using interventions that are efficacious, well tolerated, and safe. Given the difficulty with measuring fatigue, which relies heavily on patient self-report, studies on MS-associated fatigue to date have been limited compared with its prevalence. Given that fatigue is the most commonly reported symptom among pMS, and has only a few medication options, including complex options such as controlled stimulant medications, a nonpharmacological option is timely. Given the rise of several disease-modifying therapies for MS, and the panoply of tolerability and safety issues that accompany them, symptomatic therapies for pMS outside of disease modification have value. If studied in a rigorous fashion, interventions for fatigue in MS may be shown to provide important data for fatigue levels and patterns in people with MS, reduce self-reported fatigue, improve quality of life, and provide a new line of investigation for understanding the fundamental pathophysiology of fatigue in a chronic progressive brain disorder.

LT is also a low-cost option compared with many medications. The LT box costs several hundred US dollars and would be expected to last for months to years. Compared with the cost of medications, visits to the pharmacy, and related covered and out-of-pocket expenses, LT confers a distinct cost benefit to pMS. When considered from a wider societal perspective, LT would be further presumed to be cost-effective because a reduction in the fatigue level of pMS would increase their productivity at work, at school, and during other activities, allowing them to contribute to their environments and avert disability.

We here provide a study design protocol for researching the effects of LT on adults with MS-associated fatigue. Recruitment of participants who suffer from severe fatigue is very feasible given that fatigue is highly prevalent throughout the disease course of MS, affecting people with both relapsing remitting and progressive subtypes of the disease. Given a clear lack of suitable treatment options available and patients’ own reluctance to take additional medications, LT may be a welcomed addition to a therapeutic regimen. Conversely, if LT is not efficacious or well tolerated among pMS, exploration of the reasons for its inefficacy and the subgroups with particular benefits or challenges will ultimately provide valuable insights. The MS patient community has both celebrated and suffered from a variety of nonpharmacological options for their disease in recent years. In some cases, patient enthusiasm, but limited evidence, has led to high costs but no value to patients’ clinical outcomes. The MS community thus deserves the highest quality of evidence upon which to make treatment decisions and spend their time, money, and effort. We include our screening methods, inclusion and exclusion criteria, and procedural details for enrollment, evaluation, and analyses for future researchers who may wish to replicate, expand, or modify their own LT or MS-associated fatigue studies.

The study contains limitations related to the light box equipment and the logistical issues of a self-administered intervention requiring frequent participant self-report in a relapsing condition. The light boxes used in the study are large and somewhat cumbersome, which might prevent participants from bringing the light boxes with them throughout the day when necessary to complete the two 1-hour sessions, resulting in missed sessions. To minimize this limitation, we provide working participants with 2 light boxes, one that can be left in the office and one that can be left at home. MS most often affects the working-age population, and scheduling daily LT interventions around work could be difficult or problematic for some participants. This could result in participants failing to adhere to a consistent treatment schedule as directed. In addition, it is difficult to monitor participant adherence to the LT treatment, and thus, the self-reported data might not accurately reflect light box usage. Although our sample size is designed and powered to meet our primary endpoint, we recognize that 15 points on the FSS is a fairly high number and larger sample sizes would allow a more detailed understanding of more modest effects and subgroups of interest (e.g., progressive vs relapsing remitting MS, younger vs older, etc.), as well as minimize the risk of a type II statistical error in inference.

The effect of the season on fatigue levels is a potential confounding factor in this study, as participants might naturally have greater light exposure in the summer than in the winter in the New England area. However, the effect of seasonality cannot be determined given this study design. We are also exploring the impact of vitamin D levels in the serum during the study, but have no a priori hypothesis that the level will be related to the therapeutic response to LT.

Another potential limitation is comorbid conditions that may also be contributing to fatigue, although we have attempted to eliminate these confounders with our exclusion criteria and baseline screening questionnaires.

In summary, we believe that LT has several important features that may make it a valuable symptomatic treatment for MS-associated fatigue, if our results show therapeutic benefit. The results of the trial will be published in full in a peer-reviewed medical journal for dissemination to patients, clinicians, policy makers, and other stakeholders upon study completion. Further research on the use of LT in different groups of MS patients, such as children or patients with varying comorbidities, would provide valuable information on the use of LT across the range of pMS.
